# Pharmaceutical Point of View on Parenteral Nutrition

**DOI:** 10.1155/2013/415310

**Published:** 2013-12-22

**Authors:** M. Stawny, R. Olijarczyk, E. Jaroszkiewicz, A. Jelińska

**Affiliations:** ^1^Department of Pharmaceutical Chemistry, Poznan University of Medical Sciences, 6 Grunwaldzka, 60-780 Poznań, Poland; ^2^School of Pharmacy, De Montfort University, The Gateway, Leicester LE19 BH, UK

## Abstract

Parenteral nutrition—a form of administering nutrients, electrolytes, trace elements, vitamins, and water—is a widely used mode of therapy applied in many diseases, in patients of different ages both at home and in hospital. The success of nutritional therapy depends chiefly on proper determination of the patient's energetic and electrolytic needs as well as preparation and administration of a safe nutritional mixture. As a parenterally administered drug, it is expected to be microbiologically and physicochemically stable, with all of the components compatible with each other. It is very difficult to obtain a stable nutritional mixture due to the fact that it is a complex, two-phase drug. Also, the risk of incompatibility between mixture components and packaging should be taken into consideration and possibly eliminated. Since parenteral nutrition is a part of therapy, simultaneous use of drugs may cause pharmacokinetic and pharmacodynamic interactions as well as those with the pharmaceutical phase. The aim of this paper is to discuss such aspects of parenteral nutrition as mixture stability, methodology, and methods for determining the stability of nutritional mixtures and drugs added to them.

## 1. Introduction

Parenteral nutrition entered clinical practice in the late 1960s and was one of the most important developments in medicine after antisepsis, anesthesia, and antibiotics. Currently, parenteral nutrition is a generally accepted and accessible way of treatment when gastrointestinal system is either inefficient, totally nonfunctional, or inaccessible [1].

Parenteral nutrition is a way of delivering, in the form of intravenous infusion, the nourishments necessary for the maintenance of life, such as amino acids—a source of proteins, glucose, and lipids—a supply of energy; and water, electrolytes, microelements, and vitamins [2].

Parenteral nutrition is used primarily in therapies of gastrointestinal patients after stomach resection, with short bowel syndrome, intestinal fistula, bowel obstruction, and absorption disorders (Crohn's disease, acute pancreatitis) and as perioperative treatment in malnourished or depleted patients with extensive burns, and those in shock and during chemo- and radiotherapy [3].

There are three conditions that have to be met in order to provide successful parenteral nutrition. The amounts of nutrients required for life maintenance must cover the metabolic and energetic needs of the patient. The proportion between the content of nitrogen and the energy supplied by nutrients other than proteins (1 g of N should be equivalent to 130–200 kcal) and the ratio between the carbohydrates and lipids (50–75 kcal from carbohydrates per 25–50 kcal from lipids) have to be correct to ensure appropriate use of the components in the biochemical processes of the body. Also, parenteral nutrition is expected to be individualized by addressing not only the patient's energetic and electrolytic needs but also his or her general health status, coexisting illnesses, and emotional state [2].

From the pharmaceutical point of view, nutritional mixture is a complex, double-phase, and sterile drug. Such definition of the nutritional mixture helps to avoid incompatibilities between the ingredients and to maintain its physicochemical and microbiological stability. Considering the profile of a parenterally nourished patient, it must be remembered that parenteral nutrition may not be the only medical intervention applied in a given case. It is common that parenteral nutrition plays only a support part in the treatment of an underlying disease that involves pharmacotherapy, often polypragmasy, which may cause severe interactions not only between the drugs being used but also between parenteral mixture ingredients [4].

## 2. Mixture Stability in Parenteral Nutrition

Parenteral nutrition is a multicomponent medication. With about fifty components coexisting in one container and the possibility of interactions between them, the container, and excipients, it is probably the most complex therapy in modern medicine [4, 5]. Tables 1 and 2 show the contents of amino acids and lipids in emulsion preparations—the most complex components of parenteral nutrition.

The most important interactions in the aqueous phase are as follows.

1. *Precipitation of Calcium Hydrogen Phosphate*. The sediment is deposited when the product of concentration of Ca^2+^ and HPO_4_^2−^ ions is above 72 mmol^2^/L^2^. Many other factors such as pH and the content of the mixture, the way it was prepared, and storage conditions may affect the solubility of CaHPO_4_. Currently, the risk of precipitation of CaHPO_4_ can be eliminated by the use of organic calcium salts such as gluconates and glycerophosphate which do not dissociate in aqueous solutions [6].
2. *Inactivation of Vitamins, as They Are Highly Susceptible to Degradation*. Parenteral nutrition generally contains vitamins at the minimal concentrations necessary for the body function. Sometimes the clinical state of the patient requires additional supplementation with high doses of some vitamins: vitamin B_1_ in severe malnutrition or vitamin C in patients with increased cell catabolism. Inactivation of vitamins may follow many mechanisms: photolysis of vitamin A and B_1_, oxidation of vitamin C, reduction of vitamin B_1_, or adsorption of vitamin A onto the surface of the container [6].

Changes occurring in the lipid phase are equally dangerous. Parenteral nutrition is a two-phase medication consisting of o/w emulsion, formed as a result of mixing the aqueous (amino acids, carbohydrates, electrolytes, and microelements) and the lipid phases.

Lipid emulsions used in parenteral nutrition consist of spherical particles of lipids of micellar structure and size comparable to chylomicrons. External part of micelles is hydrophilic with a negative charge while on the inside lipophilic chains of fatty acids are present. The negative charge localized on the external part of the micelles prevents their aggregation, which can be the first sign of the degradation of lipid emulsions. The biggest danger to the stability of micelles is the presence of di- or trivalent cations, which can neutralize the negative charge on micelles leading to the formation of larger particles. Therefore, a crucial parameter characterizing parenteral nutrition is the critical aggregation number (CAN)—a maximal concentration of cations above which the aggregation of lipid particles can occur [5, 7].

The literature presents two solutions to ensure the stability of parenteral nutrition. One of them is based on the stability of components and chosen drugs in parenteral nutrition without the lipid emulsions. Parenteral nutrition mixtures, so-called 2 in 1, containing carbohydrates, amino acids, electrolytes, and trace elements, are used most frequently in parenteral nutrition for neonatals and pediatric patients. In these cases, lipid emulsions with vitamins are administered from a separate container and mixed with aqueous phase at the vein access. The reason for this is a high concentration of cations, above the CAN, which could lead to the degradation of lipid emulsions in the parenteral nutrition mixtures [5].

The other solution relies on the stability of the complete parenteral nutrition mixture, which is much difficult to investigate due to the biphasic character of the mixture.

## 3. Methods of Parenteral Nutrition Analysis

When approaching the problem of parenteral nutrition stability, one has to consider the complexity of the mixture and analytical problems connected with the assessment of its stability. Despite the wide range of analytical techniques available, there are no methods for quick determination of the stability of parenteral nutrition. It is therefore extremely important to design an experiment and choose a technique which will allow to determine all critical factors that might influence its stability.

The majority of studies were carried out on parenteral nutrition stored between 24 hours and several weeks, in temperature ranges of 2–8°C and 36–38°C, and with various degrees of exposure to light [8, 9].

The influence of packaging on the stability of parenteral nutrition mixture was also studied [10, 11]. It was found that storage time and temperature as well as exposure to light had the greatest effect on the stability of parenteral nutrition mixtures. The following was observed: increased decomposition of vit. A in the presence of sunlight, decreased decomposition of vitamins at low temperatures, changes in colour during storage at room temperature, decreased decomposition of vitamins in multilayered packaging, phase separation of lipid emulsion during storage, while their content appeared to have a lesser effect on their stability. The type of amino acid and lipid emulsion, the concentration of electrolytes, and the addition of antioxidant vitamins were also observed to affect the stability of parenteral nutrition mixtures [9–23].

At present, international regulations do not indicate the most suitable methodology for studying the stability of parenteral nutrition mixtures or provide standards for their physicochemical safety.

The stability of parenteral nutrition mixtures is generally investigated in three areas:

1. stability of lipid emulsion and the effect of other components on its degradation,
2. parameters characterizing the properties of parenteral nutrition mixtures,
3. stability of the components.

Organoleptic, in particular visual assessment, can give vital information on the stability of parenteral nutrition. Since lipid emulsion is a very sensitive component, the degradation of emulsion frequently manifests itself by the separation of phases. Color change during storage may also be an indication of degradation whereas the measurement of pH, osmolarity, and electrical conductance offer information with regard to initial changes in parenteral nutrition mixture [7].

As the lack of any changes is not synonymous with the stability of parenteral nutrition mixtures, further investigations are necessary. The emulsion should be examined for any changes in particle size, signs of lipid peroxidation, and factors that may cause or affect it. In the aqueous phase the concentrations of glucose, amino acids, and electrolytes need to be monitored and if any loss occurs, identification of degradation products and their biological properties is necessary.

Lipid emulsions used in parenteral nutrition are o/w emulsions, having a narrow pH range and low osmolarity, which allows them to be given via peripheral intravenous access. The stability of lipid emulsion is a resultant of attractive forces, mainly of van der Waals character and repulsive forces (electrostatic and spherical) which act on lipid emulsion particles. Electrostatic forces, being dependent on the pH, concentration of electrolytes, and the presence of surfactants, have the largest effect as they prevent emulsion particles from forming aggregates [21]. One of the best parameters to characterize the stability of lipid emulsion particles is potential zeta, an electric potential formed just outside the Stern layer. This potential determines the strength of electrostatic interaction between the particles [21]. Zeta potential can be established with the help of the Doppler effect during electrophoresis (Laser Doppler Electrophoresis (LDE)) or via an electrophoretic light scattering experiment (Electrophoretic Light Scattering (ELS)).

The stability of lipid emulsions can be studied by measuring particle sizes using optical microscopy, laser diffraction, or dynamic light scattering. These methods were used successfully in the determination of size and shape of emulsion particles and their stability [12–15, 22].

Atomic absorption spectroscopy (AAS) and inductively coupled plasma atomic emission spectroscopy (ICP-AES) are the most suitable techniques to determine metal ion concentrations in parenteral nutrition [24]. So far, only one investigation using ICP-AES and ICP-MS in the study of metal ions (Na and K ions) in parenteral nutrition was reported [25]. Antes et al. [26] applied ICP-MS to detect the level of metals in raw materials used to produce parenteral nutrition, such as solutions of amino acids, glucose, and electrolytes. The authors identified and assayed a number of heavy metals present in the analyzed matrices such as cadmium, mercury, copper, and manganese and found their concentrations exceeding the limit levels (Table 3).

Other methods reported more frequently for the analysis of metal ions were flame photometry, capillary electrophoresis, and ion-selective electrodes, but the results obtained may carry large errors because of the complexity of the matrices [27–29].

The most common methods for glucose assays are enzymatic assays using glucose oxidase or hexokinase. In the first method, glucose is oxidized to gluconic acid and hydrogen peroxide is formed. Hydrogen peroxide in the presence of peroxidase reacts with the sodium salt of 2,2′-azino-di-(3-ethyl-benzthiazoline-6-sulphonic acid) to give a colored product, absorbing in the range 670–680 nm [30]. The second relies on phosphorylation of glucose in the presence of hexokinase and ATP and formation of glucose-6-phosphate and ADP. Glucose-6-phosphate is oxidized to 6-phosphogluconate and NAD is reduced to NADH. Formation of NADH is associated with increased absorbance at 340 nm [31]. Both assays can be done directly, without any initial extractions, using commercial tests. The hexokinase method was used successfully by Bouchoud et al. [11]. If an assay indicates a lowered concentration of glucose in the parenteral nutrition than expected, a reaction between some amino acid (lysine, histidine, or cysteine) and glucose can be suspected. The reaction between these substrates, known as Maillard reaction, leads to the formation of unstable N-substituted glycosylamine, which then degrades forming smaller molecules containing an active subgroup O=C–C–N. According to Fry and Stegink [32], the Mallard reaction is affected not only by the type of amino acid but also the pH of the mixture, temperature, and the presence of electrolytes (Table 4).

HPLC methods can be used to determine concentration of amino acids in parenteral nutrition mixture which do not contain any lipid emulsions. In all-in-one mixtures separation of aqueous and lipid phases is necessary. Solid phase extraction (SPE) is predominant because analyte loss is low, in contrast to classical extraction, and it is effective and simple to perform [33].

## 4. Drug Stability in Parenteral Nutrition

HPLC methods were used successfully in assays of vitamins in parenteral nutrition mixtures, especially in parenteral nutrition free from lipid emulsion. Vazquez et al. [34] designed a method that assayed 13 vitamins in one experiment using HPLC-UV-MS-MS. The need for complex tandem spectrometry resulted from the low selectivity of the UV detector and lack of a suitable chromophore in some of the vitamins assayed, for example, in pantothenic acid. Ribeiro et al. [23] assayed vitamins B_1_ and B_6_ using an HPLC method with diode array detection, vitamin B_2_ utilizing fluorometry, and vitamin C by means of iodometric titration. HPLC methods were also used to assay cocarboxylase [35], vitamin A and E [19], and dehydroascorbic acid [36]. Baumgartner et al. [37], Iqbal et al. [33], and Wade et al. [38] designed a method for chromatographic separation and assay of vitamin B_1_ and ranitidine in two-in-one mixtures [37], cefepime [33], and ceftazidime [38]. This type of research, although extremely important and carrying high clinical potential, is rarely undertaken because of the complexity of analytical methods.

Parenteral nutrition is frequently only one of many therapies used and patients are likely to be given other drugs as well. These drugs, which as a result of primary or coexisting disease cannot be delivered via gastrointestinal system, must be given intravenously either as an injection or infusion. That results in the patient being given additional liquids either as an isotonic salt solution or solution of glucose leading to either hypernatraemia or hyperglycaemia. Adding drugs to parenteral nutrition may eliminate this problem and reduce the cost of pharmacotherapy but one has to ensure the following:

1. adequate drug stability by considering the effect of parenteral nutrition on drug stability and the impact of the drug on parenteral nutrition stability and
2. desired pharmacokinetic parameters during the long time infusion in order to maintain the proper drug concentration in the blood [4, 39].

At the moment, parenterally fed patients receive most of indispensable drugs administered in the same way, through vascular access with a Y-connector. The procedure minimalizes but does not entirely eliminate the contact between the drug and the components of the nutrition mixture. In some cases (Table 5) even a short-term contact between the drug and the nutrition mixture can cause precipitation, color change, or phase separation. That is the reason why before administering the drug alongside the nutritional mixture using the Y-connector, it must be assured that no interaction in the pharmaceutical phase will occur [39].  Table 6 summarizes drugs compatible with the NuTRIflex Lipid Special mixture [40]. Some schemes for administering antibiotics simultaneously with parenteral nutrition are presented below [39–42]:

- ampicillin: infusion of not more than 20 minutes,
- ceftazidime: infusion of not more than 30 minutes,
- ciprofloxacin: infusion of not more than 30 minutes,
- imipenem/cilastatin: fast infusion (10–15 min); stop supply of parenteral nutrition, rinse vascular access before and after infusion,
- vancomycin: stop supply of parenteral nutrition, rinse vascular access before and after infusion.

A simultaneous supply of drugs and parenteral nutrition may cause not only interactions in the pharmaceutical phase, but also pharmacokinetic and pharmacodynamic interactions resulting in a drug protein binding degree change, a cytochrome P450 activity modification, or an alteration in the distribution volume of drugs through a modified flow of the extracelluar fluid. Such incompatibilities are difficult to predict and have not been adequately researched [39].

## 5. Conclusion

Parenteral nutrition should involve administration of a fully balanced nutritional mixture covering patients' energetic and water-electrolyte needs, resulting in an improved health status, life maintenance, or quality life enhancement.

The success of parenteral nutrition therapy depends on preparation and administration of a safe nutritional mixture—a physicochemically and microbiologically stable drug. The preparation of such mixture should be preceded by analysing its composition and any interactions which might occur during preparation, storage, and infusion. It is equally important to develop and validate a method for determining mixture stability. Stability analysis ought to be performed before adding drugs to the nutritional mixture and its administration via intravenous access.

Therefore, the role of the pharmacist should ensure the therapeutic safety of parenteral nutrition in all its aspects including parenteral nutrition mixture preparation, choice of an appropriate administration route and drug form for the ongoing medication, implementation of alternative treatment methods, monitoring therapeutic and toxic effects, and instructing the medical and nursing staff about possible interactions of drugs with parenteral nutrition.

## Figures and Tables

**Table 1:** Composition of some amino acid solutions.

	Aminoplasmal 10%	Aminoplasmal HEPA	Aminomel 10%	Aminosteril 10%	Aminosteril N-Hepa	Aminoven infant 10%	Nephrotect	Primene 10%	Vamin 14	Vaminolact
	[g/1000 mL]									
Isoleucine	8.9	8.8	5.85	4.67	10.4	8.0	5.8	6.7	4.2	3.1
Leucine	5.0	13.6	6.24	7.06	13.09	13.0	12.8	10.0	5.9	7.0
Lysine acetate		10.6	10.02		9.71		16.9			
Methionine	4.4	1.2	4.68	4.1	1.1	3.12	2	2.4	4.2	1.3
Phenylalanine	4.7	1.6	5.4	4.82	0.88	3.75	3.5	4.2	5.9	2.7
Threonine	4.2	4.6	5.0	4.21	4.4	4.4	8.2	3.7	4.2	3.6
Tryptophan	1.6	1.5	2.0	1.82	0.7	2.01	3.0	2.0	1.4	1.4
Valine	6.2	10.6	5.0	5.92	10.08	9	8.7	7.6	5.5	3.6
Arginine	11.5	8.8	9.66	10.64	10.72	7.5	8.2	8.4	8.4	4.1
Histidine	3.0	4.7	3.3	2.88	2.8	4.76	9.8	3.8	5.1	2.1
Glycine	12.0	6.3	7.55	15.95	5.82	4.15	5.31	4.0	5.9	2.1
Alanine	10.5	8.3	15.5	15.0	4.64	9.3	6.2	8.0	12	6.3
Proline	5.5	7.1	7.5	15.0	5.73	9.71	3.0	3.0	5.1	5.6
Aspartic acid	5.6	2.5	1.91					6.0	2.5	4.1
Asparagine		0.55								
Acetylcysteine		0.8	0.67		0.7		0.54			
Glutamic acid	7.2	5.7	5.0					10	4.2	7.1
Serine	2.3	3.7	4.3		2.24	7.67	7.6	4.0	3.4	3.8
Acetyl tyrosine		0.86	2.0							
Tyrosine	0.4					4.2	0.6	0.45	0.17	0.5
Lysine	8.56			7.46		8.51		11	6.8	5.6
Taurine						0.4		0.6		0.3
Cysteine						0.52		2.46	0.42	1.0
N-glycyl-L-tyrosine							3.16			
Ornithine		1.66	2.42					2.49		
Cystine									0.42	1.0

**Table 2:** Composition of some lipid emulsion preparations.

	ClinOleic 20%	Intralipid 10%	Intralipid 20%	Intralipid 30%	Lipofundin MCT/LCT 10%	Lipofundin MCT/LCT 20%	Lipofundin N 10%	Lipofundin N 20%	SMOFlipid
	[g/1000 mL]								
Olive oil	160								50
Soybean oil	40	100	200	300	50	100	100	200	60
Medium-chain triglycerides					50	100			60
Lecithin from egg yolk					8	12	8	12	
Glycerol					25	25	25	25	
Fish oil									30

**Table 3:** Content of heavy metals in raw materials used in production of parenteral nutrition [26].

Element	Certified value	Amino acids	Glucose	Lipids
	[*µ*g/kg]			
As	26.67 ± 0.41	26.2 ± 1.3	27.2 ± 1.2	26.9 ± 1.2
Cd	22.79 ± 0.96	22.8 ± 1.2	23.1 ± 1.1	22.9 ± 0.8
Pb	27.89 ± 0.14	27.0 ± 1.4	27.7 ± 1.3	27.8 ± 1.2
Hg*	1.590 ± 0.018	1.57 ± 0.07	1.56 ± 0.06	1.61 ± 0.08
Cr	38.6 ± 1.6	37.5 ± 1.9	38.2 ± 1.6	37.9 ± 1.3
Cu	85.2 ± 1.2	87.5 ± 3.9	86.2 ± 4.2	84.3 ± 4.1
Mn	121.5 ± 1.1	120 ± 5.1	120 ± 6.0	123 ± 5.8
Mo	46.75 ± 0.26	46.8 ± 2.2	46.0 ± 2.2	47.4 ± 2.3
Ni	27.40 ± 0.80	27.0 ± 1.3	26.7 ± 1.3	27.3 ± 1.1
V	12.99 ± 0.37	12.9 ± 0.6	13.2 ± 0.6	12.9 ± 0.5

*Results are in [mg/kg].

**Table 4:** Percent of amino acid forming Maillard reaction products [32].

Amino acid	Time [day]	Storage temperature					
		4°C	4°C + electrolytes	25°C	25°C + electrolytes	60°C	60°C + electrolytes
		[%]					
Valine	1	0.00	0.00	0.03	0.06	1.68	1.65
	7	0.00	0.00	0.29	0.98	—	—
	30	0.00	0.00	0.40	1.27	—	—

Isoleucine	1	0.00	0.00	0.03	0.06	10.8	13
	7	0.00	0.00	0.68	0.86	—	—
	30	0.00	0.00	2.30	4.79	—	—

Leucine	1	0.08	0.12	0.06	0.30	10.1	12.1
	7	0.32	0.41	0.18	0.19	—	—
	30	0.70	1.07	1.00	1.73	—	—

Proline	1	0.00	0.00	0.01	0.01	0.31	0.48
	7	0.00	0.00	0.15	0.19	—	—
	30	0.00	0.01	0.40	0.53	—	—

Phenylalanine	1	0.00	0.00	0.02	0.06	7.46	7.86
	7	0.00	0.00	0.57	0.84	—	—
	30	0.00	0.01	0.64	2.56	—	—

Lysine	1	0.00	0.00	0.33	0.92	10.9	11.2
	7	0.00	0.30	1.76	3.61	—	—
	30	0.00	0.50	2.60	10.5	—	—

Arginine	1	0.09	0.10	1.00	1.48	9.61	15.2
	7	0.61	1.06	1.80	2.89	—	—
	30	0.96	5.61	3.96	9.61	—	—

Alanine	1	0.11	0.79	0.43	0.74	4.61	4.54
	7	0.16	1.01	0.75	1.03	—	—
	30	0.81	0.91	1.45	1.41	—	—

Glycine	1	0.50	1.61	0.82	1.38	4.75	7.97
	7	0.52	1.54	2.12	3.33	—	—
	30	1.74	2.55	3.59	6.71	—	—

Methionine	1	0.19	0.42	1.81	4.60	10.9	13.5
	7	1.10	2.00	3.90	8.90	—	—
	30	2.81	3.82	8.41	14.7	—	—

Histidine	1	0.10	0.43	0.16	1.50	16.4	31.1
	7	0.22	0.44	0.42	3.38	—	—
	30	0.30	0.82	1.50	13.0	—	—

Serine	1	0.90	1.10	1.51	2.60	8.51	13.9
	7	1.25	1.50	4.52	4.87	—	—
	30	3.20	5.40	9.61	12.2	—	—

Threonine	1	0.53	1.43	1.11	2.11	9.67	15.5
	7	0.97	1.56	4.01	4.88	—	—
	30	2.52	6.04	8.82	9.87	—	—

Tryptophan	1	2.29	4.33	4.02	5.44	6.60	12.1
	7	3.42	5.21	11.1	11.1	—	—
	30	8.14	13.6	15.8	21.1	—	—

**Table 5:** Examples of drugs incompatible with parenteral nutrition [39].

Drugs incompatible with parenteral nutrition		
Aciclovir	Ganciclovir Na	Methyldopate HCl
Amphotericin B	Haloperidol	Midazolam HCl
Ciclosporine	Heparin	Minocycline HCl
Dopamine HCl	Hydrochloric acid	Nalbuphine HCl
Doxorubicin	Hydromorphone	Ondansetron HCl
Doxycycline hyclate	Iron dextran	Pentobarbital Na
Droperidol	Levorphanol tartrate	Phenobarbital Na
Fluorouracil	Lorazepam	Phenytoin Na

**Table 6:** Drugs compatible with NuTRIflex Lipid Special after 1 hour contact mixed in 1 : 1 (v/v) proportions [40].

Drug	Dose	Drug	Dose
Calcium chloride	0.13 mmol/mL Ca	Midazolam	2.5 mg/mL
Cefepime	100 mg/mL	Morphine sulfate	5 mg/mL
Cyclosporine	2.5 mg/mL	Noradrenaline	1 mg/mL
Fentanyl	0.05 mg/mL	Octreotide	25 *μ*g/mL
Furosemide	10 mg/mL	Ondansetron	2 mg/mL
Tropisetron	1 mg/mL	Paracetamol	10 mg/mL
Magnesium sulfate	0.4 mmol/mL Mg	Piperacillin/Tazobactam	80 mg/mL (piperacillin)
Meropenem	50 mg/mL	Potassium phosphate	0.12 mmol/L PO_4_
Metoclopramide	5 mg/mL	Tacrolimus	0.1 mg/mL
Metronidazole	5 mg/mL	Vancomycin	10 mg/mL

Composition of NuTRIflex Lipid Special (B.Braun Medical) per 1000 mL: amino acids 57.4 g; Lipids 40 g; glucose 144 g; sodium 54 mmol; potassium 38 mmol; calcium 4.2 mmol; magnesium 4.2 mmol; phosphate 16 mmol; chloride 48 mmol; Acetate 48 mmol; zinc 0.03 mmol.
